# Clinical impact of the PAI-1 4G/5G polymorphism in Chinese patients with venous thromboembolism

**DOI:** 10.1186/s12959-022-00430-x

**Published:** 2022-11-14

**Authors:** Ziran Wang, Lingjun Kong, Guoju Luo, Han Zhang, Fengchun Sun, Wenjuan Liang, Wei Wu, Zijian Guo, Rui Zhang, Yaling Dou

**Affiliations:** 1 Department of Clinical Laboratory, Peking Union Medical College Hospital, Chinese Academy of Medical Sciences, Beijing, China; 2 Department of Clinical Laboratory, Affiliated Hospital of Jining Medical University, Shandong Province, Jining, China; 3 Medical Laboratory Center, Northwest Women’s and Children’s Hospital, Shaxi Province, Xi’an, China

**Keywords:** **Venous thromboembolism**, **PAI-1**, **Gene polymorphism**

## Abstract

**Background:**

Venous thromboembolism (VTE) is a life-threatening cardiovascular syndrome that characterized by the imbalance of hemostasis and thrombosis and the formation of thrombi in the blood vessels. The aim of this study was to elucidate the clinical impact of the PAI-1 4G/5G polymorphism in Chinese patients with VTE.

**Methods:**

A total of 169 subjects (89 VTE, 10 hyperbilirubinemia, 10 hyperlipidemia and 60 healthy controls) were recruited at Peking Union Medical College Hospital. The accuracy of the TaqMan-MGB RT-PCR method for detecting *F5* G1691A (FVL) and PAI-1 4G/5G polymorphisms was evaluated by using sequencing method as the gold standard. Besides, the association of the PAI-1 4G/5G polymorphism with susceptibility, treatment efficacy and recurrence status of VTE in Chinese population were explored. Eventually, the plasma PAI-1 antigen levels and PAI-1 4G/5G polymorphisms were determined on additional 64 subjects (32 VTE and 32 healthy controls) simultaneously.

**Results:**

The TaqMan-MGB RT-PCR method was proven to be highly accurate in determining the FVL and PAI-1 4G/5G polymorphisms without interference from bilirubin and lipids in the samples. No obvious correlation of the PAI-1 4G/5G polymorphism with VTE was observed in our study by using five genetic models (allele, genotype, dominant, recessive and additive). Additionally, we also observed that individuals with the 4G/5G genotype had lower neutrophil counts and neutrophil-to-lymphocyte ratio (NLR) than the 5G/5G genotype. Furthermore, we found that the patients with the 5G/5G genotype were more likely to achieve complete recanalization compared to the 4G/4G genotype. In addition, individuals carrying the 5G/5G genotype were more likely to develop a recurrence-free status as compared to individuals with the 4G/4G or 4G/5G genotypes. PAI-1 antigen levels in the VTE group were significantly higher than those in the HC group. However, there was no significant difference in the antigen levels of PAI-1 among subjects carrying various genotypes in the VTE group or HC group.

**Conclusion:**

The PAI-1 4G/5G polymorphism has potential value in assessing the prognosis of Chinese patients with VTE. Our study has laid the foundation for the application of PAI-1 4G/5G polymorphism in the personalized management and monitoring of patients with VTE.

## Introduction

Venous thromboembolism (VTE), including deep-vein thrombosis (DVT) and pulmonary embolism (PE), is the third most common acute cardiovascular syndrome and its disease burden is growing as the population and life expectancy expand [1]. DVT is defined as venous thrombus forming in a large vein, such as the leg or pelvis, while PE is developed when the thrombus dislodges and spreads through the heart to the pulmonary arteries [2]. VTE has become a global public health concern, with an estimated 300,000–600,000 individuals affected each year in the United States, offering high morbidity and mortality rates [3]. Notably, patients with coronavirus disease 2019 (COVID-19) were proven to be at significantly higher risk of developing VTE due to concomitant prothrombotic status [4].

The etiology of VTE is complicated and varied, and its onset may be attributed to the disruption of the coagulation homeostasis. Several factors may be involved in the development of VTE, including intrinsic (e.g., thrombophilia), acquired (e.g., obesity, cancer, prothrombotic medication) and external (e.g., reduced mobility due to surgery, trips lasting more than four hours) [5]. For patients with suspected VTE, it is recommended to combine clinical pretest probability assessment, D-dimer test, and imaging for diagnosis [6]. Patients with VTE would benefit from prompt anticoagulation treatment based on bleeding risk assessment. Different pharmaceutical regimens such as heparin, low-molecular-weight heparin (LMWH), fondaparinux, or the direct oral anticoagulants (rivaroxaban or apixaban) should be applied on the basis of the patient's individual condition and duration of treatment [7]. However, given the aggressive nature of VTE episodes, early risk assessment and refined management for patients would be imperative. In this scenario, it is meaningful to explore individualized biomarkers for the meticulous management of VTE patients.

Given the crucial role of genetic factors in disease pathogenesis, single nucleotide polymorphisms (SNPs) have become a hot topic of research at present. In previous study, we reported the potential value of combining tumor necrosis factor-α (*TNF-α*) -308G/A gene polymorphism with neutrophil-to-lymphocyte ratio (NLR) and platelet-to-lymphocyte ratio (PLR) in predicting the efficacy and safety of anti-TNF-α therapy [8]. Some inherited polymorphisms have been identified as high-risk factors for VTE, such as 4G or 5G polymorphism of plasminogen activator inhibitor 1 (PAI-1), G1691A mutation in the *F5* gene (Factor V Leiden (FVL)), G20210A of the *F2* (prothrombin) gene, and C677T of the methylenetetrahydrofolate reductase (MTHFR) gene.

PAI-1 gene (*SERPINE1*) is located on chromosome 7q which encodes the secreted protein containing 402 amino acids [9]. As the primary inhibitor of tissue-type and urokinase-type plasminogen activator, PAI-1 is an integral member of the fibrinolysis system [10]. Abnormally increased PAI-1 can impair plasminogen activation resulting in excessive fibrin accumulation in the blood vessels, which further leads to thrombosis. A single nucleotide insertion or deletion (4G or 5G) polymorphism at 675 bp upstream of the PAI-1 gene (*SERPINE1*) transcription start site has been reported to be associated with the expression of PAI-1, with the 4G allele being favored for elevated expression levels [11, 12]. The 4G/5G polymorphism of the PAI-1 has been reported to be associated with the risk of venous thrombosis [13], ischemic stroke [14], femoral necrosis [15], diabetic nephropathy [16], cancers [17], and systemic lupus erythematosus [18]. Unfortunately, the association of the PAI-1 4G/5G polymorphism with susceptibility, treatment efficacy and recurrence status of VTE has not been well investigated, especially in Chinese population.

Factor V is a crucial component involved in the coagulation process and it can be cleaved at amino acid 506 by activated protein C (APC). However, when the nucleotide at position 1691 of the *F5* gene is converted from guanine to adenine, the arginine at position 506 is substituted by glutamine, which is known as the FVL mutation. In this case, APC fails to cleave factor V and thus blocks the anticoagulant effect. It has been reported that FVL heterozygote and homozygote carriers have a sevenfold and 80-fold increased risk of VTE, respectively, relative to individuals without FVL mutation [19]. Notably, FVL appears to be relatively rare in Asian populations, particularly in the Chinese population [20]. However, given the tremendous risk of thrombosis associated with FVL mutation, the assessment of FVL genotypes was also included in this study. In addition, there was a report claiming that FVL may interact with the PAI-1 4G/5G polymorphism to determine the risk of recurrence of VTE in a Swedish population [21]. On this basis, the roles of the PAI-1 4G/5G and FVL polymorphisms in Chinese VTE patients were desired to be explored in this study.

Thus, we evaluated the accuracy of TaqMan-minor groove binder (MGB) reverse transcription-polymerase chain reaction (RT-PCR) method for detecting PAI-1 4G/5G and FVL polymorphism using sequencing method as the gold standard. Besides, we also provided a new perspective on the potential utility of the PAI-1 4G/5G polymorphism in relation to VTE risk, treatment efficacy and recurrence status in Chinese population.

## Materials and methods

### Subjects

A total of 169 subjects (89 VTE, 10 hyperbilirubinemia, 10 hyperlipidemia and 60 healthy controls) were recruited at Peking Union Medical College Hospital (PUMCH), Chinese Academy of Medical Sciences (CAMS) between July 2019 to September 2020. The diagnosis of VTE was based on the patients' clinical presentation, laboratory finding (D-dimer) and imaging studies such as ultrasonography, computed tomography pulmonary angiography (CTPA) or lung ventilation-perfusion scan [6]. The status of the vessels after treatment was divided into the following categories: [1] complete recanalization, with unobstructed blood flow in the vessels and no residual thrombus; [2] partial recanalization, with residual thrombus narrowing the vessels or a small interruption of continuous blood flow in at least one previously thrombosed vessel; [3] no recanalization, interruption of blood flow in the vessels which previously had the thrombus [22]. Recurrence of VTE was defined as: [1] dissemination of thrombus to new veins or venous segments; [2] formation of new thrombus at a different site; [3] new PE [23]. Hyperbilirubinemia (HB) and hyperlipidemia (HLP) were diagnosed by combining clinical symptoms and laboratory results as described in the previous publication [8]. In addition to this, apparently healthy individuals with no family history of coagulation disorders, no malignancies, rheumatic or chronic diseases and no abnormal laboratory test results were selected as healthy controls (HC). Duplicate individuals were removed using unique ID codes. Eventually, the plasma PAI-1 antigen levels and PAI-1 4G/5G polymorphisms were determined on additional 64 subjects (32 VTE and 32 HC) simultaneously in August 2022. The diagnostic criteria for VTE and the inclusion criteria for HC are consistent with those described above.

### Ethics statement

This study was conducted in accordance with the recommendations of the PUMCH. All procedures performed in studies involving human participants were in accordance with the ethical standards of the institutional and/or national research committee with the 1964 Helsinki declaration and its later amendments or comparable ethical standards. This study was approved by the Ethics Committee of PUMCH (No. HS2019036).

### Sample preparation and data collection

Sample collection, processing and results verification were carried out in accordance with standard operating procedures. Venous whole blood samples were collected from the subjects and anticoagulated with EDTA-K2, then whole blood cells were sorted and counted using XN-9100 (Sysmex Corporation, Japan). NLR is calculated by dividing the neutrophil count by the lymphocyte count while PLR is obtained by dividing the platelet count by the lymphocyte count. For the coagulation test, 109 mmol/L of sodium citrate was mixed thoroughly with venous whole blood at 1:9 and then centrifuged at 3000 g for 15 min at room temperature. The obtained plasma was tested using the CS-5100 instrument (Sysmex Corporation, Japan). Data regarding the subject's baseline information, medical records, laboratory results, imaging findings, drug consumption records, treatment efficacy and recurrence status were accessed through the hospital information system (HIS) and laboratory information system (LIS) of PUMCH.

### Genotyping

Genomic DNA was extracted from EDTA-K2 anticoagulated venous whole blood using the Tianlong extraction reagents and matching instruments (Tianlong Technology Co. LTD, Xi’an, China). The FVL and PAI-1 4G/5G genotypes of individuals were determined using the TaqMan-MGB RT-PCR detection kit (Wuhan HealthCare Biotechnology Co. LTD, Wuhan, China) in Applied Biosystems (ABI) 7500 Real-time System (ABI Inc. CA, United States). The primer and probe sequences were shown in Table 1. The PCR reaction volume is 50 μl per well, including 5 μl of PCR reaction solution (primers and probes), 25 μl of PCR Mix and 20 μl of genomic DNA. The RT-PCR reaction procedure can be divided into three stages: the first stage is pre-denaturation at 95 °C for 1 min; the second stage is 95 °C for 5 s and 61 °C for 32 s with a total of 15 cycles; the third stage is 95 °C for 5 s and 61 °C for 32 s (fluorescence collection) with a total of 30 cycles. After the PCR procedure was completed, the results were analyzed using the ABI 7500 system software. For the FVL polymorphism, the VIC, FAM and ROX channels are used to detect the amplified fluorescent signals of the G and A alleles and β-actin (internal control) respectively. For the PAI-1 4G/5G polymorphism, the VIC, FAM and ROX channels are used to detect the amplified fluorescent signals of the 5G and 4G alleles and β-actin respectively. The positive result is defined as a smooth S-shaped amplification curve with a Ct value < 17 and ∆Rn > 100,000. Meanwhile, genotyping was also performed by using the ABI Real-time Prism 3730XL Sequence Detection System (ABI Inc. CA, United States) according to the Applied Biosystem protocol.

**Table 1:** Primer and probe sequences for the detection of PAI-1 and FVL polymorphisms

Targets	Sequences
**PAI-1 4G/5G**	
Forward primer	5'-GCCAGACAAGGTTGTTGACACA-3'
Reverse primer	5'-GAGGACTCTTGGTCTTTCCCTCAT-3'
5G allele probe	5'-VIC-CTGACTCCCCCACGTG-MGB-NFQ-3'
4G allele probe	5'-FAM-CTGACTCCCCACGTGT-MGB-NFQ-3'
***F5* G1691A (FVL)**	
Forward primer	5'-GACCATACTACAGTGACGTGGACATC-3'
Reverse primer	5'-CCCATTATTTAGCCAGGAGACCTAAC-3'
G allele probe	5'-VIC-TATTCCTCGCCTGTCCA-MGB-NFQ-3'
A allele probe	5'-FAM-ACCTGTATTCCTTGCCTG-MGB-NFQ-3'
***β-Actin***	
Forward primer	5'-TGAAGATCCTCACCGAGCG-3'
Reverse primer	5'-GGCAGCTCGTAGCTCTTCTC-3'
Probe	5'-ROX-ACCACCACGGCCGAGCGG-BHQ-3'

### Plasma PAI-1 antigen levels measurement

Plasma PAI-1 antigen levels were measured by using the PAI-1 ELISA kit (CUSABIO Biotechnology Co. LTD, Wuhan, China). The assay range of PAI-1 for this kit is 3.125–200 ng/ml. The kit is for research use only and no reference range has been established for healthy control subjects. The test was performed in accordance with the manufacturer’s recommendations. Briefly, EDTA-anticoagulated whole blood was centrifuged at 1000 × g for 15 min and the supernatant separated and stored at -20 °C. Prior to the initiation of the experiment, eight gradient standards at concentrations of 0, 3.125, 6.25, 12.5, 25, 50, 100 and 200 ng/ml were obtained by diluting the standard (200 ng/ml) in the kit. Add 100 μl of plasma or standards to the 96-well plates and incubate for 2 h at 37 °C, then discard the liquid and add the biotin-labeled antibody and incubate for 1 h at 37 °C. After washing, 100 μl of affinity-labeled horseradish peroxidase was added to each well and incubated for 1 h at 37 °C. Finally, 90 μl of the substrate was added to each well for incubation away from light and the absorbance was measured at 450 nm. The standard curve was fitted based on the absorbance and concentration of the eight standards. The antigen levels of PAI-1 for each sample were determined by comparing its absorbance with the standard curve.

#### Statistical analysis

All data was documented, calculated and analyzed using Excel 2019 (Microsoft Inc., United States), MedCalc software (version 20.0), R Project (version 4.2.0) and RStudio (Open-Source Edition) software. The t-test or Wilcoxon test was used for analysis of continuous data while the chi-square test or Fisher's exact probability for analysis of categorical data. The concordance between genotyping methods was analyzed by using kappa test. The Hardy–Weinberg equilibrium (HWE) test was performed on the genotype frequency distribution. Logistic regression or logistic regression adjusted for age and sex was used to determine the association of various genotypes/alleles of the PAI-1 4G/5G polymorphism with VTE susceptibility. The odds ratios (ORs) and 95% confidence intervals (CIs) were calculated in the different genetic models. *P* < 0.05 was considered statistically significant.

## Results

### Methodological evaluation of genotype determination

**Fig. 1:**
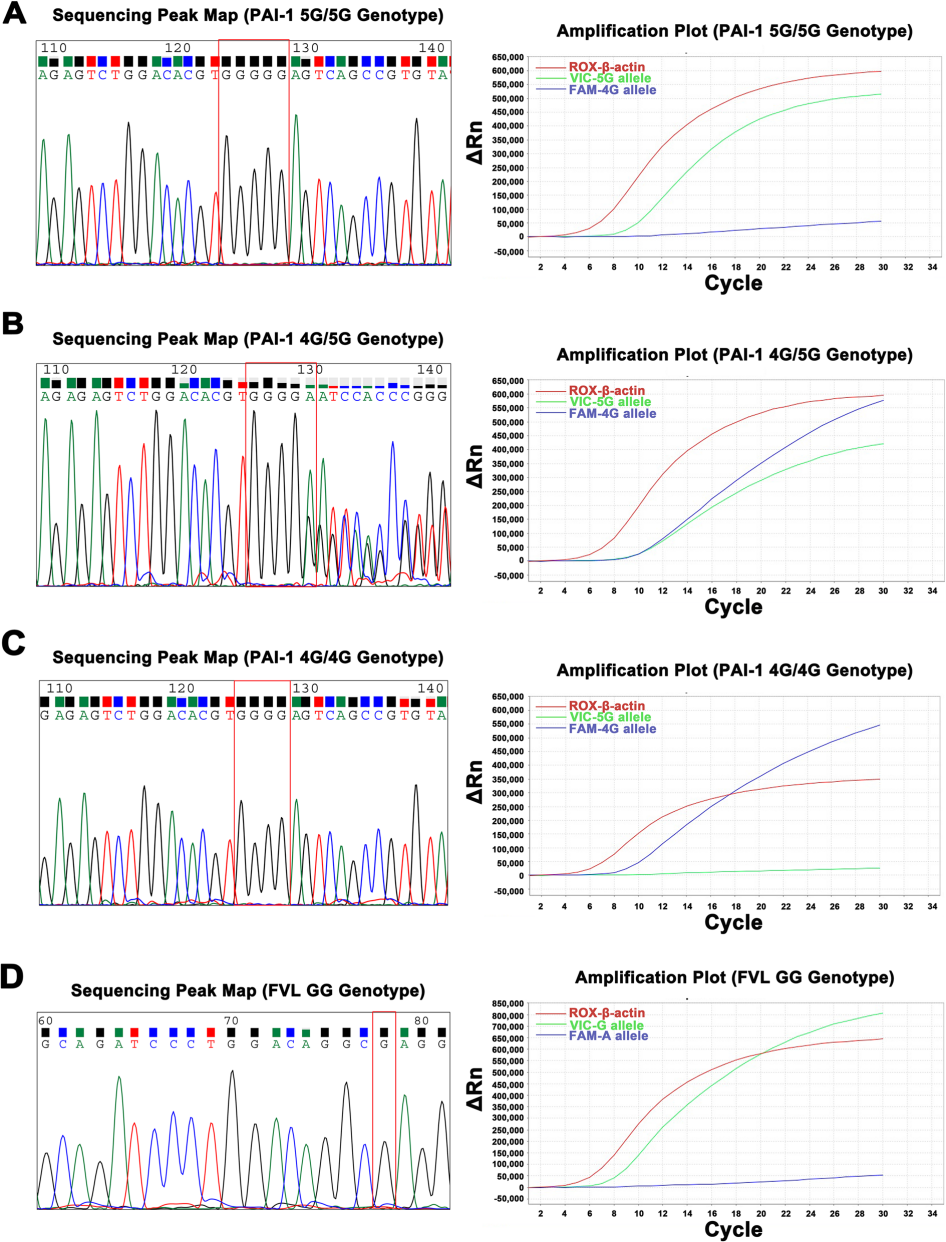
Sequencing peak maps and amplification plots of FVL and PAI-1 4G/5G polymorphisms **A**, 5G/5G genotype of PAI-1; **B**, 4G/5G genotype of PAI-1; **C**, 4G/4G genotype of PAI-1; **D**, GG genotype of FVL

In this study, we evaluated the accuracy of TaqMan-MGB RT-PCR method for the identification of FVL and PAI-1 4G/5G polymorphisms, using sequencing method as the gold standard. A total of 169 subjects (89 VTE, 10 HB, 10 HLP and 60 HC) were enrolled. For the FVL polymorphism, the TaqMan-MGB RT-PCR method and the sequencing method gave consistent results (Table 2). Meanwhile, the results of both methods were identical in HB and HLP patients, indicating that bilirubin and lipids do not affect the TaqMan-MGB RT-PCR procedure. Intriguingly, all individuals were identified as GG wild type and no other genotypes were observed for FVL (Fig. 1A). For the PAI-1 4G/5G polymorphism, the TaqMan-MGB RT-PCR method also showed a high degree of concordance compared to the sequencing method and was not interfered with by bilirubin or lipids (Table 3). Specifically, in the VTE group, 35 were 4G/4G genotype, 36 were 4G/5G genotype and 18 were 5G/5G genotype, while in the HC group, 16 were 4G/4G genotype, 36 were 4G/5G genotype and 8 were 5G/5G genotype. The sequencing peak maps and amplification plots for the 4G/4G, 4G/5G and 5G/5G genotypes were presented in Fig. 1B-D. Overall, the TaqMan-MGB RT-PCR method was proven to be highly accurate in determining the FVL and PAI-1 4G/5G polymorphisms without interference from bilirubin and lipids in the samples.

**Table 2:** Detection of the genotype of FVL by sequencing and TaqMan-MGB RT-PCR

**Group**	**Number of cases**	**Sequencing**			**TaqMan-MGB RT-PCR**			**Kappa value**	***P* value**
		GG	GA	AA	GG	GA	AA		
VTE	89	89	0	0	89	0	0	1	< 0.001
HC	60	60	0	0	60	0	0	1	< 0.001
HB	10	10	0	0	10	0	0	1	< 0.001
HLP	10	10	0	0	10	0	0	1	< 0.001

*VTE* Venous thromboembolism, *HC* Healthy controls, *HB* Hyperbilirubinemia, *HLP* Hyperlipidemia

**Table 3:** Detection of the genotype of PAI-1 4G/5G by sequencing and TaqMan-MGB RT-PCR

**Group**	**Number of cases**	**Sequencing**			**TaqMan-MGB RT-PCR**			**Kappa value**	***P* value**
		5G/5G	4G/5G	4G/4G	5G/5G	4G/5G	4G/4G		
VTE	89	18	36	35	18	36	35	1	< 0.001
HC	60	8	36	16	8	36	16	1	< 0.001
HB	10	2	4	4	2	4	4	1	< 0.001
HLP	10	3	5	2	3	5	2	1	< 0.001

*VTE* Venous thromboembolism, *HC* Healthy controls, *HB* Hyperbilirubinemia, *HLP* Hyperlipidemia

### PAI-1 4G/5G polymorphism and VTE risk

As we observed, no FVL mutation was detected in all subjects in the VTE and HC groups. Given the rarity of FVL mutation in Chinese population [24], we excluded it from the follow-up study.

The clinical baseline characteristics of the VTE and HC groups were shown in Table 4. Compared to the HC group, subjects in the VTE group were older and more male. In terms of laboratory parameters, the VTE group had higher white blood cell counts, neutrophil counts and NLR, and lower lymphocyte counts. The HWE test showed that the PAI-1 4G/5G genotype frequencies fulfilled the law of genetic equilibrium (*P* > 0.05). To explore the association between the PAI-1 4G/5G polymorphism and VTE risk, five genetic models (allele, genotype, dominant, recessive and additive) were performed. As shown in Table 5, no correlation was observed between the PAI-1 4G/5G polymorphism and VTE risk based on allele model (4G vs 5G, OR = 1.126, 95%CI = 0.704–1.800, *P* = 0.6206), genotype model (4G/5G vs 5G/5G, OR = 0.444, 95%CI = 0.172–1.152, *P* = 0.0872; 4G/4G vs 5G/5G, OR = 0.972, 95%CI = 0.350–2.700, *P* = 0.9569), dominant model (4G/5G + 4G/4G vs 5G/5G, OR = 0.607, 95%CI = 0.245–1.502, *P* = 0.2707), recessive model (4G/4G vs 4G/5G + 5G/5G, OR = 1.782, 95%CI = 0.874–3.636, *P* = 0.107) and additive model (OR = 1.125, 95%CI = 0.705–1.797, *P* = 0.6215). The correlation between the PAI-1 4G/5G polymorphism and VTE risk in the five models was still not found after adjusting for age and sex (Table 5).

**Table 4:** The clinical baseline characteristics of the VTE and HC groups

Variables	Total (*n* = 149)	VTE (*n* = 89)	HC (*n* = 60)	*P* value
Age, Median (IQR)	42 (32, 57)	53 (40, 67)	34 (31, 39)	** < 0.001**
Sex, n (%)				** < 0.001**
F	90 (60)	42 (47)	48 (80)	
M	59 (40)	47 (53)	12 (20)	
FVL genotype, n (%)				1
GG	149 (100)	89 (100)	60 (100)	
PAI-1 genotype, n (%)				0.064
4G/4G	51 (34)	35 (39)	16 (27)	
4G/5G	72 (48)	36 (40)	36 (60)	
5G/5G	26 (17)	18 (20)	8 (13)	
RBC, × 10^12^/L, Median (IQR)	4.36 (4.08, 4.69)	4.33 (3.91, 4.77)	4.41 (4.23, 4.63)	0.207
Hb, g/L, Median (IQR)	134 (122, 144)	133.5 (117, 146)	134.5 (126, 142.25)	0.314
HCT, %, Mean ± SD	38.8 ± 4.85	38.72 ± 5.85	38.92 ± 2.83	0.778
WBC, × 10^9^/L, Median (IQR)	5.95 (5.15, 7.28)	6.54 (5.3, 8.95)	5.77 (4.87, 6.25)	**0.001**
L, × 10^9^/L, Mean ± SD	1.62 ± 0.55	1.56 ± 0.63	1.72 ± 0.38	**0.049**
N, × 10^9^/L, Median (IQR)	3.7 (2.91, 5.11)	4.16 (3.15, 6.47)	3.51 (2.77, 3.87)	** < 0.001**
PLT, × 10^9^/L, Median (IQR)	218 (187.75, 260.75)	211.5 (160.75, 276)	224.5 (197.75, 256)	0.197
NLR, Median (IQR)	2.15 (1.7, 3.2)	2.59 (1.92, 4.8)	1.99 (1.61, 2.34)	** < 0.001**
PLR, Median (IQR)	138.23 (104.54, 177.2)	139.86 (98.16, 202.84)	135.53 (111.71, 164.29)	0.362

*VTE* Venous thromboembolism, *HC* Healthy controls, *RBC* Red blood cell counts, *Hb* Hemoglobin, *HCT* Hematocrit, *WBC* White blood cell counts *L* Lymphocyte counts, *N* Neutrophil counts, *PLT* Platelet counts, *NLR* Neutrophil-to-lymphocyte ratio, *PLR* Platelet-to-lymphocyte ratio, *IQR* Interquartile range, *SD* Standard deviation

**Table 5:** Analysis of the association between the PAI-1 4G/5G polymorphism and VTE risk

Model	Cases	Controls	*P* value	OR	95% CI	Adjusted^a^ *P* value	Adjusted^a^ OR	Adjusted^a^ 95% CI
**Allele**								
5G	72	52		1	-			
4G	106	68	0.6206	1.126	0.704–1.800	0.545	1.201	0.664–2.171
**Genotype**								
5G/5G	18	8		1	-			
4G/5G	36	36	0.0872	0.444	0.172–1.152	0.8545	1.132	0.301–4.266
4G/4G	35	16	0.9569	0.972	0.350–2.700	0.7433	1.223	0.366–4.084
**Dominant model**								
5G/5G	18	8		1	-			
4G/5G + 4G/4G	71	52	0.2707	0.607	0.245–1.502	0.8645	1.105	0.351–3.480
**Recessive model**								
4G/5G + 5G/5G	54	44		1	-			
4G/4G	35	16	0.107	1.782	0.874–3.636	0.4414	1.415	0.585–3.424
**Additive model**								
5G/5G	18	8	0.6215	1.125	0.705–1.797	0.5318	1.215	0.660–2.240
4G/5G	36	36						
4G/4G	35	16						

*VTE* Venous thromboembolism, *HC* Healthy control, *RBC* Red blood cell counts, *Hb* Hemoglobin, *HCT* Hematocrit, *WBC* White blood cell counts, *L* Lymphocyte counts, *N* Neutrophil counts, *PLT* Platelet counts, *NLR* Neutrophil-to-lymphocyte ratio, *PLR* Platelet-to-lymphocyte ratio, *IQR* Interquartile range, *SD* Standard deviation, *OR* Odds ratio *CI* Confidence interval, *a* Adjust for age and sex

### PAI-1 4G/5G polymorphism and laboratory parameters

**Fig. 2:**
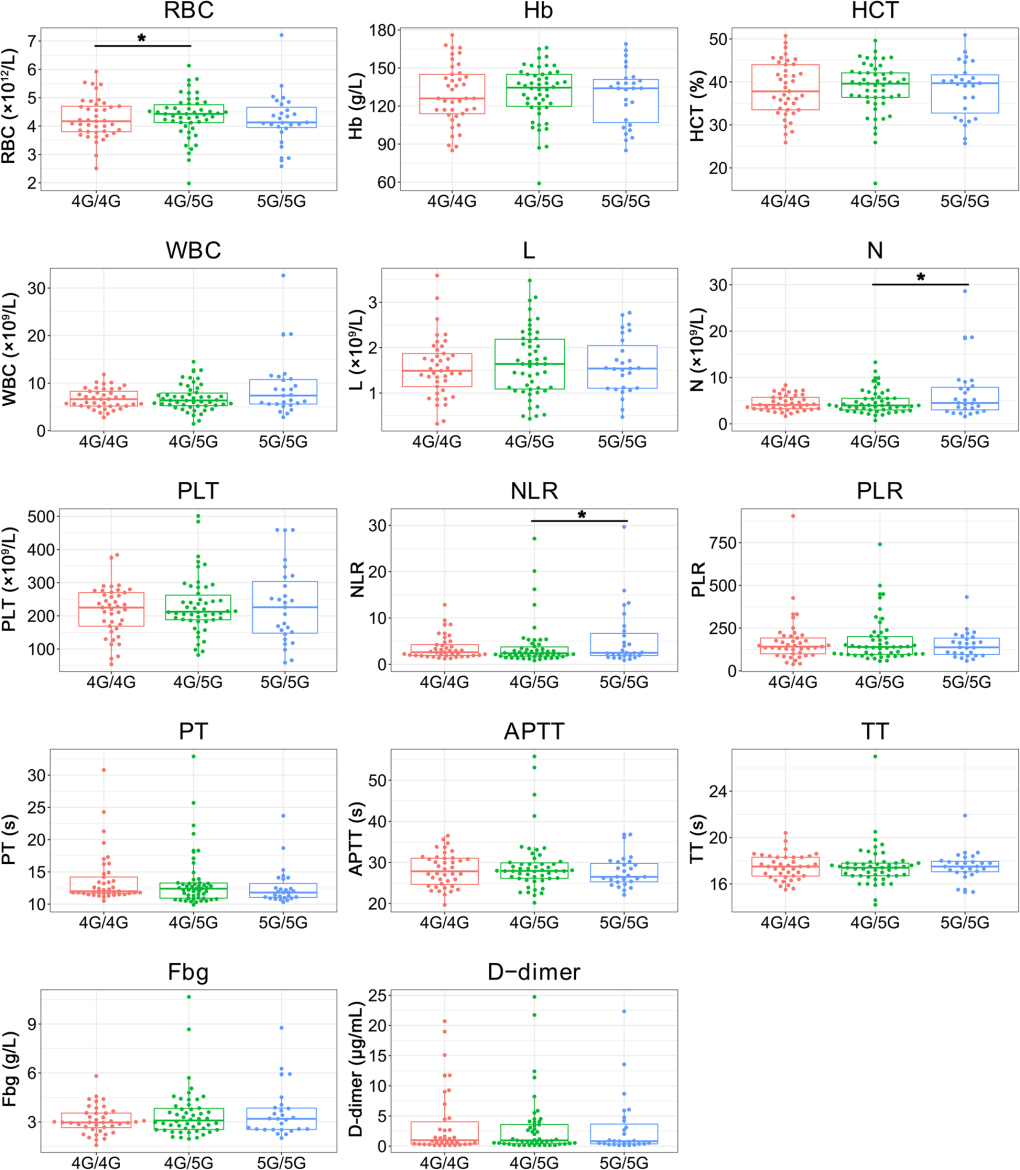
Differences in laboratory parameters among VTE patients with various genotypes of PAI-1 RBC, red blood cell counts; Hb, hemoglobin; HCT, hematocrit; WBC, white blood cell counts; L, lymphocyte counts; N, neutrophil counts; PLT, platelet counts; NLR, neutrophil-to-lymphocyte ratio; PLR, platelet-to-lymphocyte ratio; PT, prothrombin time; APTT, activated partial thromboplastin time; TT, thrombin time; Fbg, fibrinogen *P < 0.05

To explore the correlation between the PAI-1 4G/5G polymorphism and laboratory parameters, hematological markers [red blood cell counts (RBC); hemoglobin (Hb); hematocrit (HCT); white blood cell counts (WBC); lymphocyte counts (L); neutrophil counts (N); platelet counts (PLT); NLR; PLR; prothrombin time (PT); activated partial thromboplastin time (APTT); thrombin time (TT); fibrinogen (Fbg); D-dimer] of VTE patients with different genotypes were measured and compared. As illustrated in Fig. 2, individuals with the 4G/4G genotype had lower RBC counts than individuals with the 4G/5G genotype while individuals with the 4G/5G genotype had lower neutrophil counts and NLR compared to 5G/5G. Notably, the difference in RBC counts between individuals with the 4G/4G genotype and the 4G/5G genotype was no longer significant after using multiple regression analysis which adjusted for age and gender (*P* = 0.074). For other laboratory parameters, no evident differences were observed among subjects of the three genotypes.

### PAI-1 4G/5G polymorphism and treatment efficacy

Information on the treatment and follow-up of patients with VTE was recorded in Table 6. The most common site of thrombosis in this study was lower extremity DVT (64/89. 71.9%). The management of VTE was based on anticoagulation therapy, with rivaroxaban (39, 43.8%), heparin (25, 28.1%) and warfarin (19, 21.3%) being the common agents. Treatment efficacy was documented in a total of 41 patients, of whom 13 (31.7%) achieved complete recanalization, 19 (46.3%) achieved partial recanalization and 9 (22.0%) had no recanalization. In terms of the distribution of PAI-1 gene polymorphisms, eight were 5G/5G genotype, 13 were 4G/5G genotype, while the remaining 20 were 4G/4G genotype. We further compared the differences in laboratory parameters and treatment outcomes between individuals with the 4G/5G genotype and the 5G/5G genotype (Table 7). Individuals with the 4G/5G genotype had higher Hb compared to individuals with the 5G/5G genotype (*P* = 0.026), whereas other indicators were not statistically different in the two groups. Furthermore, the result of multiple regression analysis adjusted for gender and age showed that the difference in Hb between patients with the 4G/5G genotype and 5G/5G genotype was still significant (*P* = 0.028). Interestingly, when we compared the differences between individuals with the 4G/4G genotype and 5G/5G genotype (Table 7), we found that the 4G/4G genotype group had lower proportions of complete recanalization (4G/4G vs 5G/5G: 15% vs 62%, *P* = 0.031). However, no difference in complete recanalization rate was observed between the 4G/4G and 4G/5G + 5G/5G subgroups (*P* = 0.074).

**Table 6:** Analysis of treatment and follow-up of VTE patients

Variables	n (%)
**Site of thrombosis (*n* = 89)**	
Lower extremity DVT	64 (71.9)
Upper extremity DVT	5 (5.6)
PE	5 (5.6)
Lower extremity DVT + PE	6 (6.7)
Portal vein	5 (5.6)
Others^**a**^	4 (4.5)
**Treatment (*n* = 89)**	
Rivaroxaban	39 (43.8)
Heparin^**b**^	25 (28.1)
Warfarin	19 (21.3)
Aescuven Forte	11 (12.4)
Others^**c**^	17 (19.1)
**Therapeutic effect (*n* = 41)**	
Complete recanalization	13 (31.7)
Partial recanalization	19 (46.3)
No recanalization	9 (22.0)
**Recurrence status after recanalization (*n* = 32)**	
Recurrence	11 (34.4)
No recurrence	21 (65.6)

*DVT* Deep-vein thrombosis, *PE* Pulmonary embolism

^a^including superficial veins of the upper extremities, mesenteric veins

^b^including low molecular weight heparin (LMWH)

^c^including t-PA, Chinese medicine, Aspirin, surgery and Dabigatran

**Table 7:** Analysis of differences in laboratory parameters and outcomes among PAI-1 genotypes

Variables	5G/5G (*n* = 8)	4G/5G (*n* = 13)	4G/4G (*n* = 20)	*P* value (4G/5G vs 5G/5G)	*P* value (4G/4G vs 5G/5G)	*P* value (4G/4G vs 4G/5G + 5G/5G)
Age, Mean ± SD	55.88 ± 17.92	50.85 ± 15.44	56.95 ± 19.65	0.522	0.891	0.462
Sex, n (%)				0.646	1	0.44
F	4 (50)	4 (31)	11 (55)			
M	4 (50)	9 (69)	9 (45)			
RBC, × 10^12^/L, Mean ± SD	3.98 ± 1.46	4.63 ± 0.49	4.07 (3.79, 4.4)	0.255	0.195	0.246
Hb, g/L, Mean ± SD	113.38 ± 26.81	140.31 ± 11.39	123.25 ± 20.58	**0.026**	0.37	0.319
HCT, %, Mean ± SD	34.4 ± 8.44	40.9 ± 3.61	36.62 ± 5.55	0.07	0.508	0.349
WBC, × 10^9^/L, Median (IQR)	8.52 (5.68, 14.02)	6.85 (5.94, 8.93)	5.66 (4.97, 8.18)	0.547	0.136	0.1
L, × 10^9^/L, Mean ± SD	1.47 ± 0.57	1.58 ± 0.56	1.49 ± 0.63	0.686	0.95	0.791
N, × 10^9^/L, Median (IQR)	6.46 (3.35, 11.79)	4.93 (3.98, 5.51)	3.87 (3.04, 5.46)	0.595	0.213	0.148
PLT, × 10^9^/L, Mean ± SD	232 ± 134.89	245.31 ± 89.42	212.4 ± 77.75	0.809	0.708	0.342
NLR, Median (IQR)	4.36 (2.36, 9.49)	2.76 (2.1, 3.84)	2.4 (2.03, 3.59)	0.414	0.258	0.372
PLR, Mean ± SD	149.62 ± 50.29	167.93 ± 61.06	133.88 (104.09, 203.83)	0.466	0.98	0.494
PT, s, Median (IQR)	12.15 (11.07, 12.85)	12 (11.5, 13.1)	12.35 (11.78, 14.75)	0.717	0.333	0.309
Fbg, g/L, Median (IQR)	3.36 (3.01, 3.99)	3.4 (2.7, 3.78)	2.97 (2.62, 3.87)	0.514	0.373	0.397
APTT, s, Median (IQR)	26.5 (25.55, 29.65)	28.9 (27.3, 29.6)	28.52 ± 3.94	0.328	0.722	0.62
TT, s, Mean ± SD	18 ± 2.07	17.83 ± 1.11	17.46 ± 1.11	0.835	0.502	0.308
D-dimer, μg/ml, Median (IQR)	2.56 (0.71, 9.89)	1.15 (0.57, 4.09)	1.3 (0.6, 3.12)	0.697	0.492	0.725
Outcome, n (%)				0.155	**0.031**	0.074
No recanalization	2 (25)	1 (8)	6 (30)			
Partial recanalization	1 (12)	7 (54)	11 (55)			
Complete recanalization	5 (62)	5 (38)	3 (15)			

*RBC* Red blood cell counts, *Hb* Hemoglobin, *HCT* Hematocrit, *WBC* White blood cell counts, *L* Lymphocyte counts, *N* Neutrophil counts, *PLT* Platelet counts, *NLR* Neutrophil-to-lymphocyte ratio, *PLR* Platelet-to-lymphocyte ratio, *PT* Prothrombin time, *APTT* Activated partial thromboplastin time, *TT* Thrombin time, *Fbg* Fibrinogen, *IQR* Interquartile range, *SD* Standard deviation

**Fig. 3:**
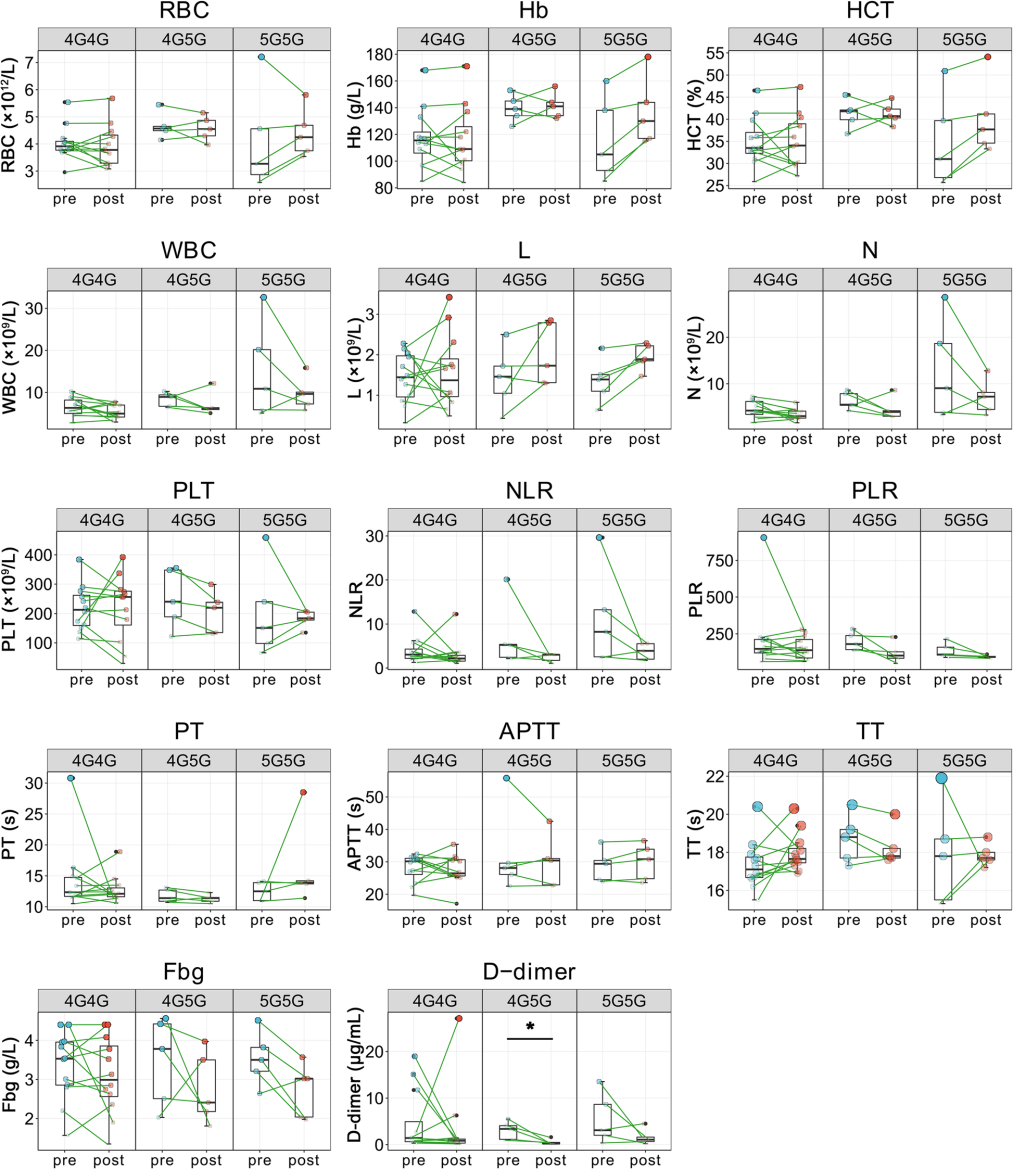
The alterations in hematological markers before and after treatment in patients with different genotypes of PAI-1 RBC, red blood cell counts; Hb, hemoglobin; HCT, hematocrit; WBC, white blood cell counts; L, lymphocyte counts; N, neutrophil counts; PLT, platelet counts; NLR, neutrophil-to-lymphocyte ratio; PLR, platelet-to-lymphocyte ratio; PT, prothrombin time; APTT, activated partial thromboplastin time; TT, thrombin time; Fbg, fibrinogen; pre, pre-treatment; post, post-treatment *P < 0.05

To further investigate the impact of various genotypes on treatment efficacy, we have analyzed the alterations in hematological markers before and after treatment in patients with different genotypes. As depicted in Fig. 3, D-dimer levels decreased after treatment in patients carrying the 4G/5G genotype whilst other laboratory parameters remained unchanged.

### PAI-1 4G/5G polymorphism and recurrence status

**Fig. 4:**
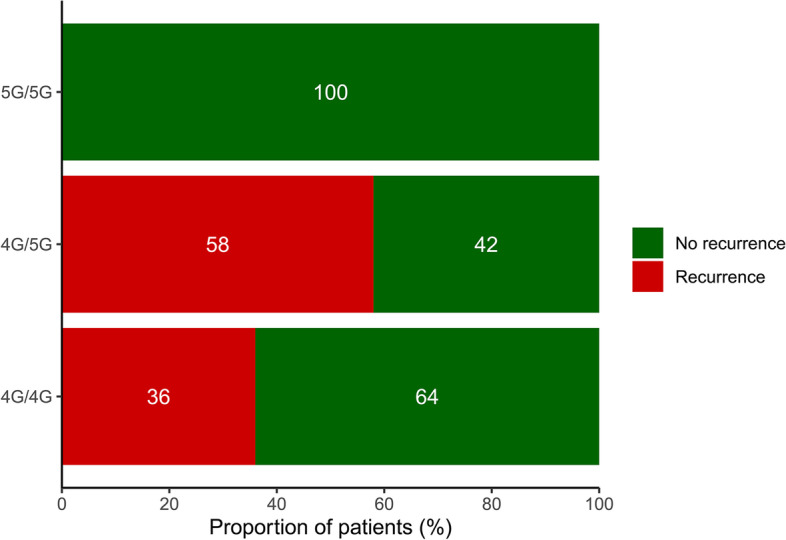
The differences in recurrence status among individuals with various genotypes of PAI-1

Follow-up of 32 VTE patients after treatment revealed that 11 had recurrence and the remaining 21 did not (Table 6). There were no variations in the type of treatment among patients with different genotypes. Consequently, we also compared the differences in recurrence status among individuals with various genotypes. As shown in Fig. 4, all individuals with the 5G/5G genotype were no recurrence while 58% and 36% of patients in the 4G/5G and 4G/4G genotype groups, respectively, developed recurrence. It can be hypothesized that individuals with the 5G/5G genotype are favored to be recurrence-free (5G/5G vs 4G/5G + 4G/4G, *P* = 0.046).

### Plasma PAI-1 antigen levels analysis

**Fig. 5:**
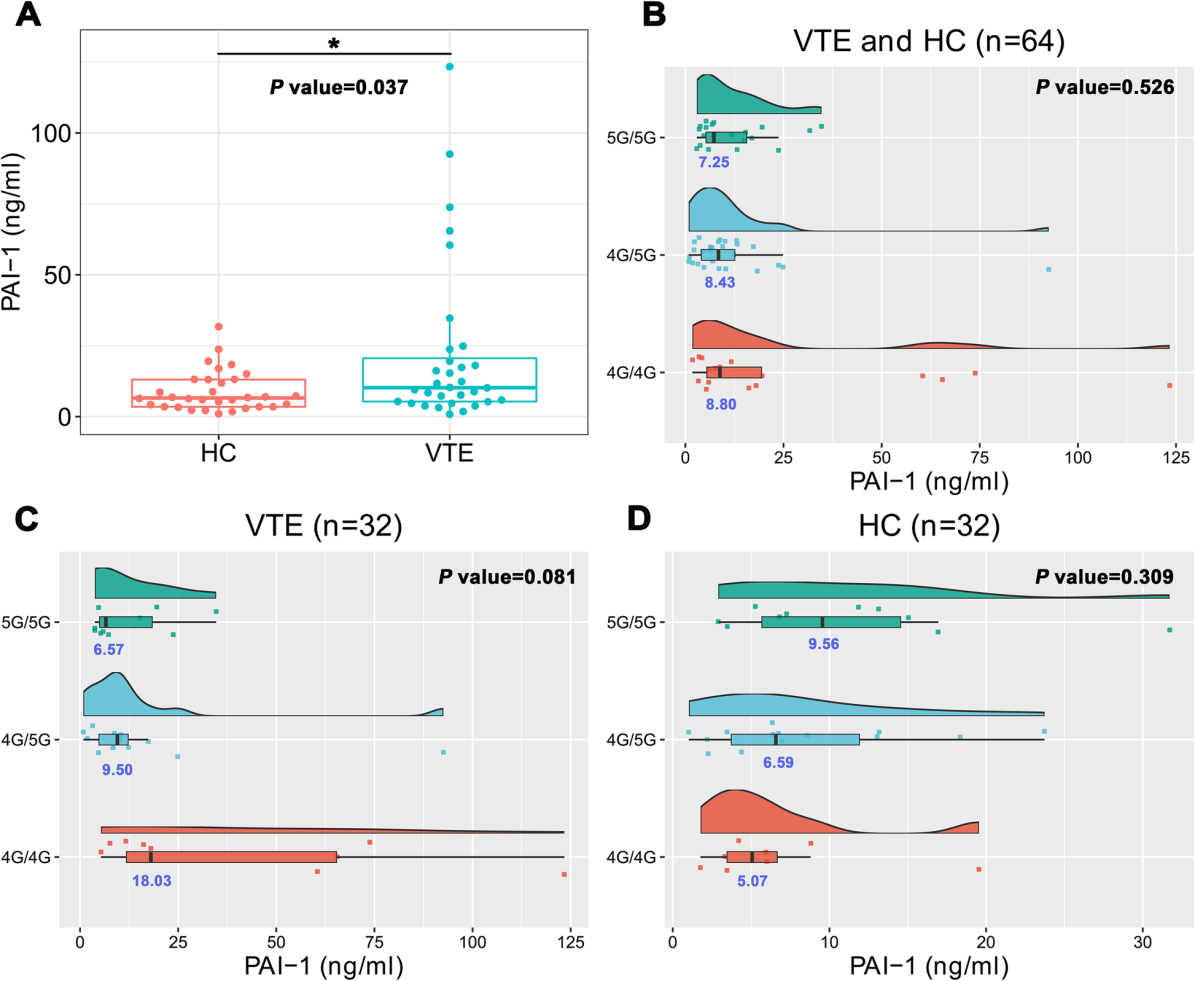
Plasma PAI-1 antigen levels analysis **A**, comparison of plasma PAI-1 antigen levels between the VTE and HC groups. **B**-**D** differences in PAI-1 plasma antigen expression levels among subjects with different PAI-1 genotypes in the VTE and HC subgroup, the VTE subgroup, and the HC subgroup, respectively. The numbers in blue below the box plots represent the median of the PAI-1 antigen levels in each group. *P < 0.05

EDTA-anticoagulated whole blood and plasma were collected from 32 VTE patients and 32 healthy controls, while PAI-1 4G/5G polymorphism and PAI-1 antigen levels were detected simultaneously. As depicted in Fig. 5A, PAI-1 antigen levels (10.21 (5.29. 22.68) ng/ml) in the VTE group were significantly higher than those (6.59 (3.48. 13.13) ng/ml) in the HC group (*P* = 0.037). Furthermore, we compared the differences in PAI-1 antigen expression levels among individuals with different PAI-1 genotypes. However, there was no significant difference in the antigen levels of PAI-1 among subjects carrying various genotypes in the VTE + HC (*P* = 0.526), VTE group (*P* = 0.081) and HC group (*P* = 0.309) (Fig. 5B-D).

## Discussion

With the development of genomics technology, the role of gene single nucleotide polymorphisms (SNPs) in the pathogenesis of disease is gradually being appreciated. Growing studies have highlighted the association of SNPs with disease susceptibility and prognosis, which is of great significance for achieving individualized medicine. VTE is closely involved with hypercoagulable status of the blood, in which genetic factors cannot be ignored. In this context, the present study focused on the association of the PAI-1 4G/5G polymorphism with VTE susceptibility, treatment efficacy and recurrence status in Chinese populations.

PAI-1 4G/5G polymorphisms have been reported in numerous studies, but there are a variety of methods for determining genotypes, including allele-specific PCR [25], TaqMan PCR [26], PCR-restriction fragment length polymorphism [27]. However, most studies have not evaluated the methodology. To ascertain the accuracy of genotype identification for FVL and PAI-1 4G/5G polymorphisms, all subjects were genotyped using sequencing (gold standard) and TaqMan-MGB RT-PCR in this study. The results showed that TaqMan-MGB RT-PCR method was highly accurate in identifying FVL and PAI-1 genotypes, consistent with the results of the sequencing method, and was not disturbed by jaundice and lipids in the samples. The TaqMan-MGB RT-PCR technique has been available for decades and is growing in development [28]. It is worth noting that the major advantages of the TaqMan-MGB probe are that it forms a stable binding to the target gene and suppresses the production of non-specific amplification products [29, 30]. Recently, TaqMan-MGB RT-PCR has been used to detect drug resistance in *Helicobacter pylori* [31], subtype analysis of swine influenza virus [32], mutation analysis of hereditary optic neuropathy [33] and SNPs detection in diabetes susceptibility genes [34]. Given that there are currently no standardized detection procedures for FVL and PAI-1 genotypes in China, our study may lay the foundation for the practical application of the TaqMan-MGB RT-PCR method in the future.

Factor V Leiden is an inherited condition that causes resistance to the anticoagulant effect of APC, which in turn increases the risk of venous thrombosis [35]. Previous research has demonstrated that FVL SNP variant (rs6025) is highly associated with the risk of VTE by using Genome Wide Association Studies (GWAS) [36]. Nevertheless, no FVL mutation was identified in all subjects in this study. Our results were consistent with the report by Wang et al. [24], implying that FVL mutation was relatively rare in Chinese population. Ethnicity appears to be an essential influence on the frequency of FVL mutation. According to a previous multiracial survey in the United States, the frequency of FVL mutation was highest in Hispanic-Americans at 1.65% and slightly lower in African-Americans at 0.87%, while no mutations were observed in Asian-Americans or Native-Americans [37].

Impairment of the fibrinolytic system plays an influential contribution in the progression of thrombotic disease. PAI-1, as the inhibitor of plasminogen, has been extensively studied. On the other hand, the role of its genetic polymorphisms in thrombotic disease has been of increasing interest. Unfortunately, the association of the PAI-1 4G/5G polymorphism with VTE susceptibility remains controversial. Many studies have proved that the PAI-1 4G/5G polymorphism was not significantly associated with VTE, while significant associations were discovered in several other studies [38]. A recent meta-analysis has shown that the PAI-1 4G/5G polymorphism was associated with an increased risk of VTE, especially in Asian populations [13]. Paradoxically, no obvious correlation of the PAI-1 4G/5G polymorphism with VTE was observed in our study by using five genetic models (allele, genotype, dominant, recessive and additive). The patient's underlying disease and risk factors may be vital points in explaining this discrepancy. Tsantes et al. reported that the association of the PAI-1 4G/5G polymorphism with VTE was evident in patients with genetic risk factors (such as family history of hereditary diseases), whereas this association was no longer elucidated in patients (such as antiphospholipid antibody syndrome, Bechet disease) which had no genetic risk factors [39]. In our study, there were no explicit genetic risk factors in the majority of cases, and the venous thrombosis developed secondary to tumors or autoimmune diseases. In addition to this, the limited sample size and the differences in ethnicity in various regions of China were not negligible elements. Therefore, future multicenter studies could contribute to a more profound understanding of the effect of the PAI-1 4G/5G polymorphism in the susceptibility to VTE in Chinese patients.

To further explore the laboratory characteristics of VTE patients with different genotypes of PAI-1, we analyzed the heterogeneity of hematological markers. Most laboratory parameters do not differ among patients with various genotypes. Patients carrying the 4G/4G genotype had lower RBC than those with the 4G/5G genotype. Considering the potential effect of gender on RBC counts, we re-evaluated this difference by performing multiple regression analysis adjusted for age and gender. It is interesting to note that this difference is no longer significant through the multiple regression analysis. Considering the limited sample size of this study, it would be desirable to conduct regional collaborative studies to clarify the effect of PAI-1 genotypes on RBC counts.

Additionally, we also observed that individuals with the 4G/5G genotype had lower neutrophil counts and NLR than the 5G/5G genotype while lymphocyte counts and PLR did not change significantly. The interaction of inflammation and coagulation suggested the subtle role of neutrophils in thrombosis. Kushnir et al. reported that persistent neutrophilia was a marker for non-malignancy, non-infected VTE patients [40]. Mechanistically, the neutrophil-derived enzymes may inhibit anticoagulant factors such as APC, antithrombin and tissue factor pathway inhibitor [41]. Furthermore, the neutrophil serine proteases and extracellular nucleosomes can enhance tissue factor- and factor XII-dependent coagulation pathways [42]. The 5G/5G genotype carriers have higher levels of neutrophils perhaps related to the fact that most of them are tumor patients, whereas tumors are thought to be closely associated with the activation of the coagulation system and the formation of thrombus. The role of neutrophils in the PAI-1 4G/5G polymorphism deserves further investigation to elucidate the complex involvement of blood cells in the coagulation, fibrinolytic and inflammatory pathways.

Currently, the management of VTE is mainly dependent on anticoagulation therapy. Nevertheless, few studies have reported the association between the PAI-1 4G/5G polymorphism and treatment efficacy. In this study, we found that the patients with the 5G/5G genotype were more likely to achieve complete recanalization compared to the 4G/4G genotype, hinting that individual with the 5G/5G genotype may be able to benefit more from treatment. Similarly, Fernandez-Cadenas et al. have reported that patients with the 4G/4G genotype had higher rates of re-occlusion compared to patients with other genotypes, heralding poor prognosis after thrombolytic therapy in patients with ischemic stroke [43]. Several studies have illustrated that the PAI-1 4G/5G polymorphism can affect the expression of PAI-1, with the 4G/4G genotype being the most highly abundant and the 5G/5G genotype being the least abundant [44, 45]. As the inhibitor of plasmin formation, high concentrations of PAI-1 may contribute to the deposition of fibrin in the vessel rather than being lysed, thus preventing complete recanalization. Another interesting finding was that individuals with the 4G/5G genotype had a reduction in D-dimer levels after treatment. D-dimer has been extensively studied as the degradation product of fibrin formation resulting from the dissolution of thrombi by the fibrinolytic system [46]. Therefore, the decrease in D-dimer may be indicative of effective treatment and thrombus lysis. Intriguingly, the D-dimer graph seems to show a decreasing trend after treatment as a whole in all genotypes. It is just that some cases are significantly increased in patients with the 4G/4G genotype and some increased in patients with the 5G/5G genotype. In this context, we have reviewed the medical records of individuals whose D-dimer did not decline after treatment. D-dimer is known to be a non-specific indicator that may be elevated in a variety of physiological and pathological conditions. We found the decline in D-dimer may be influenced by the patient's underlying diseases, the invasive nature of the treatment and the duration of bed rest.

There is a broad consensus on the critical nature of genetic factors in reoccurring thrombosis. As for the recurrent status of VTE, the PAI-1 4G/5G polymorphism also appears to be of potential utility. Our results indicated that individuals carrying the 5G/5G genotype were more likely to develop a recurrence-free status as compared to individuals with the 4G/4G or 4G/5G genotypes. The presence of the PAI-1 4G allele has been reported to increase the risk of thrombosis in patients with other thrombotic defects, such as protein C (PC) and protein S (PS) defects [47]. Similarly, another study has shown that the PAI-1 4G allele was a risk factor for the development of PE in patients with PS deficiency [48]. Thus, these studies may imply the association of the PAI-1 4G allele with the PC/PS complex deficiency. It is well known that PC, PS and phospholipids can form a complex that inactivates FVa which is considered as a pro-coagulation factor; therefore, defects in PC/PS are often closely linked to recurrent VTE [49]. We hypothesized that the relapse vulnerability of individuals carrying the 4G allele might be associated with reduced PC/PS activity. Unfortunately, studies on the correlation between PAI-1 polymorphisms and PC or PS activity levels are still scarce. Taken together, it would be meaningful to explore the association of 4G alleles with PC/PS activity levels in patients with recurrent VTE in future studies. Interestingly, the complete recanalization rate was higher in the order 5G/5G, 4G/5G, 4G/4G, which seems to be related to PAI-1 4G/5G polymorphism and the amount of PAI-1 in the blood, whilst 4G/5G was more common than 4G/4G when looking at recurrence rate. we considered that the patient's general condition, underlying disease, lifestyle habits, genetic factors and economic situation may influence the patient's prognosis and explain the above discrepancy.

When comparing the differences in PAI-1 expression levels, we found that the VTE group had relatively higher concentrations of PAI-1 than HC group. Frischmuth et al. reported that higher plasma PAI-1 levels were associated with an increased risk of future incident VTE [50]. Yang et al. suggested that plasma PAI-1 had a higher predictive value for VTE than D-dimer [51]. Thus, our results together with the above reports, may imply that plasma PAI-1 may be a potential biomarker in the diagnosis of VTE. However, when we made group comparisons by PAI-1 genotypes, no differences in plasma PAI-1 antigen levels were observed among the different subgroups in either the VTE or HC groups. Theoretically, the 4G allele could bind to the transcriptional enhancer while the 5G allele binds to the transcriptional repressor thereby resulting in higher levels of PAI-1 expression in individuals carrying the 4G/4G genotype and lower levels of PAI-1 expression in individuals with the 5G/5G genotype [13]. Nevertheless, the situation became quite diverse in terms of various diseases and populations. Chi et al. reported that individuals with the 4G/4G genotype had higher plasma PAI-1 levels relative to individuals with the 5G5G genotype in patients with severe burn sepsis [52]. In another study, the 5G/5G genotype group showed lower levels of PAI-1 compared to the 4G/4G genotype group in obese women [53]. In contrast, Sabino et al. found that young patients with ischemic stroke in Brazil had higher levels of PAI-1 compared with controls, but PAI-1 expression was not affected by the PAI-1 4G/5G polymorphism [54]. Remarkably, a study including 113 patients with rheumatoid arthritis showed that individuals with the 4G/4G genotype had higher PAI-1 mRNA levels compared to the 4G/5G or 5G/5G genotype carriers, but plasma PAI-1 levels were not significantly different [55]. Taken together, we hypothesize that PAI-1 may play an important role in the development of VTE, but its expression is not solely determined by the PAI-1 4G/5G polymorphism; factors such as patient condition, co-morbidity and ethnicity may also combine to regulate PAI-1 expression levels. Interestingly, in our study, the 4G/4G carriers appeared to have higher median PAI-1 expression levels whilst the 5G/5G carriers had lower median expression levels in the VTE group. Hence, larger sample sizes and regional comprehensive researches would be required in the future to clarify the effect of the PAI-1 4G/5G polymorphism on plasma PAI-1 expression levels in Chinese patients with VTE.

Nevertheless, there are some limitations to our study. The limited sample size necessitated caution in the interpretation of our results. However, compared to other studies, our study systematically compared the differences in the individual distribution, laboratory parameters, treatment efficacy and prognosis of the various genotypes, which could be beneficial for future applications. Future multi-center, large-scale, long dimensional studies are imperative to further delineate the role of PAI-1 4G/5G polymorphisms in VTE. Additionally, in exploring the association of the PAI-1 4G/5G polymorphism with PAI-1, we only examined the antigen expression levels of PAI-1 but not the activity of PAI-1. In the future, we would collect more samples to assess the level of PAI-1 antigen/activity in VTE patients with different PAI-1 genotypes and their changes between pre- and post-treatment. Moreover, only the roles of PAI-1 4G/5G in VTE susceptibility, treatment and prognosis were investigated in this study; other genetic mutations such as *F2* G20210A and *MTHFR* C677T will be explored in the next step. Finally, incomplete information on some patients excluded from the study may lead to biased results.

## Conclusion

Overall, our study provides new insights into the role of the PAI-1 4G/5G polymorphism in VTE. From a methodological evaluation point of view, we found that the TaqMan-MGB RT-PCR method was highly consistent with the gold standard (sequencing method) and independent of sample status, thus allowing for rapid clinical detection of PAI-1 4G/5G polymorphism. Besides, the PAI-1 4G/5G polymorphism was not associated with susceptibility to VTE in Chinese patients. Moreover, there may be variations in laboratory parameters between genotypes. Patients carrying the 4G/4G genotype had lower RBC counts than those with the 4G/5G genotype while individuals with the 4G/5G genotype had lower neutrophil counts and NLR than the 5G/5G genotype. In terms of treatment efficacy and prognosis, we found that the patients with the 5G/5G genotype was more likely to achieve complete recanalization compared to the 4G/4G genotype and individuals carrying the 5G/5G genotype were more likely to develop a recurrence-free status as compared to individuals with the 4G/4G or 4G/5G genotypes. PAI-1 antigen levels in the VTE group were significantly higher than those in the HC group. However, there was no significant difference in the antigen levels of PAI-1 among subjects carrying various genotypes in the VTE group or HC group. Consequently, our study has laid the foundation for the application of PAI-1 4G/5G polymorphism in the management and monitoring of patients with VTE.

## Data Availability

The datasets used and/or analyzed during the current study are available from the corresponding author on reasonable request.
